# Pathogenicity and immunogenicity of attenuated porcine epidemic diarrhea virus PC22A strain in conventional weaned pigs

**DOI:** 10.1186/s12917-018-1756-x

**Published:** 2019-01-11

**Authors:** Chun-Ming Lin, Shristi Ghimire, Yixuan Hou, Patricia Boley, Stephanie N. Langel, Anastasia N. Vlasova, Linda J. Saif, Qiuhong Wang

**Affiliations:** Food Animal Health Research Program, Ohio Agricultural Research and Development Center, Colleage of Food, Agricultural and Environmental Sciences, Department of Veterinary Preventive Medicine, College of Veterinary Medicine, The Ohio State University, Wooster, OH USA

**Keywords:** Porcine epidemic diarrhea virus (PEDV), Live attenuated vaccine, Mucosal immunity, Weaned pigs

## Abstract

**Background:**

Porcine epidemic diarrhea virus (PEDV) causes diarrhea in all ages of pigs with 50–100% mortality rates in neonatal piglets. In the United States, inactivated and subunit PEDV vaccines for pregnant sows are available, but fail to induce sufficient protection in neonatal piglets farrowed from PEDV naïve sows. A safe and efficacious live attenuated vaccine that can prime mucosal immune responses is urgently needed. In this study, we evaluated the safety and efficacy of two attenuated PEDV vaccine candidates, the emerging non-S INDEL PEDV strain PC22A at the 100th cell culture passage level - Clone no. 4 (P100C4) and at the 120th passage level (P120), in weaned pigs.

**Results:**

Four groups of 40-day-old weaned pigs were inoculated orally with PEDV PC22A-P3 (virulent), -P100C4, -P120, and mock, respectively, and challenged with the P3 virus at 24 days post-inoculation (dpi). After inoculation, P3 caused diarrhea in all pigs with a high level of fecal viral RNA shedding. P100C4 and P120 did not cause diarrhea in pigs, although viral RNA was detected in feces of all pigs, except for one P100C4-inoculated pig. Compared with the P120 group, P3- and P100C4-inoculated pigs had higher serum PEDV-specific IgG and viral neutralizing (VN) antibody (Ab) titers at 14 dpi. After the challenge, no pigs in the P3 group but all pigs in the P100C4, P120, and mock groups had diarrhea. Compared with the P120 group, pigs in the P100C4 group had a more rapid decline in fecal PEDV RNA shedding titers, higher titers of serum PEDV-specific IgG, IgA, and VN Abs, and higher numbers of intestinal IgA Ab-secreting cells.

**Conclusions:**

PEDV PC22A P100C4 and P120 were fully attenuated in weaned pigs but failed to elicit protection against virulent P3 challenge. P100C4 induced higher PEDV-specific antibody responses than P120 post inoculation resulting in a greater anamnestic response post challenge. Therefore, P100C4 potentially could be tested as a priming vaccine or be further modified using reverse genetics. It also can be administered in multiple doses or be combined with inactivated or subunit vaccines and adjuvants as a PEDV vaccination regimen, whose efficacy can be tested in the future.

**Electronic supplementary material:**

The online version of this article (10.1186/s12917-018-1756-x) contains supplementary material, which is available to authorized users.

## Background

Porcine epidemic diarrhea virus (PEDV), a member of the genus *Alphacoronavirus* in the family *Coronaviridae*, is highly contagious and causes enteric disease in pigs of all ages [1]. North America was free from PEDV until April 2013 when highly virulent PEDV strains, classified as non-S INDEL PEDV strains, suddenly emerged and spread quickly throughout the United States (US) [2, 3]. The major clinical signs of porcine epidemic diarrhea (PED) are diarrhea and vomiting. The mortality rates were high (50–100%) in neonatal piglets born into PEDV naïve sows [4]. Although milder clinical signs were seen in older pigs, such as weaned pigs and sows, their growth and reproductive performance were adversely impacted [5, 6]. In the US, the economic impact was the highest during 2013–2014, including the deaths of 10% of the total piglet population and $0.9–1.8 billion in losses [7]. Subsequently, the incidence of new PEDV cases showed a seasonal pattern, peaking during winter [8]. This suggests that epidemic PEDV outbreaks have decreased and PEDV infections may be mostly endemic in the US. Currently, PEDV infection occurs in at least 39 US states and some US sow herds suffer from chronic losses (https://www.aasv.org/Resources/PEDv/PEDvWhatsNew.php). Effective vaccine programs are required, but still under development [9].

It is well documented that natural infections and/or live attenuated vaccines elicit local mucosal immunity against enteric virus replication in the intestinal mucosa. In enteric virus-infected and recovered pregnant sows, gut-derived IgA immunocytes (B memory cells) migrate to the mammary glands and produce high titers of secretory IgA (sIgA) antibodies (Ab) in colostrum and milk (via the gut-mammary-sIgA axis), contributing to passive lactogenic immunity that protects neonatal suckling piglets from the disease [10, 11]. Several live attenuated PEDV vaccines, based on classical PEDV strains, such as CV777 [12], DR13 [13] and 83P-5 [14], have been used in Asia for decades. However, their protection against the emerging highly virulent PEDV strains was questionable [15, 16] because severe PED outbreaks occurred in regularly vaccinated farms. In the US, there are two conditionally licensed PEDV vaccines based on the emerging highly virulent PEDV strains, an inactivated vaccine (Zoetis, Parsippany, NJ) and an alphavirus-based PEDV subunit vaccine (Merck, Ames, IA). However, after vaccination of PEDV naïve gilts/sows, neither vaccine induced high protection rates against PED in neonatal pigs [9]. One possible reason is that intramuscular injection of inactivated or subunit PEDV vaccine mainly stimulates systemic, but not mucosal immunity [11, 17]. PEDV-specific immunity in sows recovered from previous PEDV infection can be boosted by vaccination with the inactivated vaccine to induce sufficient levels of lactogenic immunity to protect their piglets [18]. In the field, intentional infection of sows with autogenous PEDV during gestation (feedback exposure) was used to induce lactogenic immunity in sows to protect piglets [9, 19]. However, this feedback exposure has a risk of spreading other infectious agents to the entire herd and PEDV infection may become persistent on farms. Therefore, a live attenuated vaccine based on the emerging highly virulent PEDV strains that can prime mucosal immunity is urgently needed but has not been developed in the US.

In our laboratory, the original US highly virulent PEDV PC22A strain, classified as a non-S INDEL strain, was continuously passaged in Vero cells to the 160th passage (P160) [20]. PC22A P100 and the above passages showed various degrees of cell culture adaptation in vitro and virulence attenuation in vivo. Compared with the highly attenuated P160, the less attenuated P120 replicated more efficiently in neonatal piglets and induced a higher protection rate against challenge with the highly virulent homologous virus. It is well documented that weaned pigs and sows are less susceptible to PEDV infection than neonatal piglets [6, 21, 22]. Many highly attenuated PEDV/ porcine transmissible gastroenteritis virus (TGEV) strains replicated poorly and induced marginal immunity in weaned pigs and sows [23–25]. Because the evaluation of vaccine candidates in sows is very expensive and labor-intensive, young pigs, such as weaned pigs, can be used as a surrogate model to initially evaluate the safety, immune responses and protection efficacy of PEDV vaccine candidates [9]. Therefore, the aims of this study were to characterize the pathogenicity and immunogenicity of two PEDV vaccine candidates, P100C4 and P120 of the PEDV PC22A strain, in weaned pigs.

## Results

### PEDV PC22A-P100C4 and-P120 replicated in the gut but did not cause diseases in weaned pigs

**Fig. 1:**
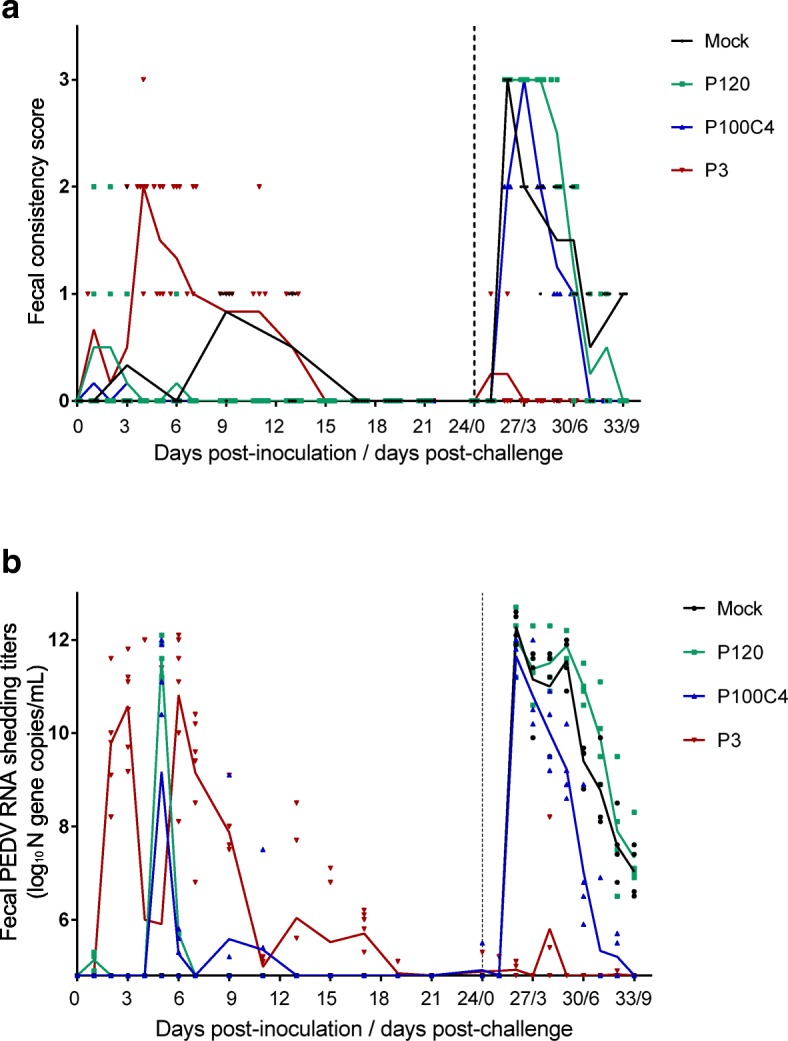
Fecal consistency (FC) score (**a**) and fecal PEDV RNA shedding titers (**b**) post inoculation and challenge. Forty-day-old weaned pigs were inoculated with mock, P100C4, P120 or P3. Subsequently, all the pigs were challenged with P3 at 24 days post-inoculation (dpi). Data are presented as mean (line) and individual data (coded symbols) in each group. The numbers of animals in each treatment group were six at inoculation and four at the challenge

All six pigs inoculated with P3 had diarrhea [fecal consistency (FC) score = 2] at 4–5 dpi and one pig had watery diarrhea (FC score = 3) for one day at 4 dpi. Diarrhea lasted for 1 to 4 days and ceased in individual pigs by 5 to 11 dpi (Fig. 1A). In addition, P3-inoculated pigs did not gain weight (− 0.30 ± 2.67 kg) during the first week (Table 1). However, the growth retardation did not last after 7 dpi. Conversely, pigs in the other groups had no clinical signs, except that transient mild diarrhea/soft feces were observed in 2 pigs around 1 to 3 dpi when fecal PEDV RNA shedding was around the detection limit or undetectable. The first-week body weight gain profiles post-inoculation were similar among pigs in the P100C4, P120, and mock groups.

**Table 1:** Clinical signs of conventional weaned pigs after PEDV inoculation at 40 days of age and challenge at 24 dpi

	After inoculation, pre-P3-challenge (1 to 21 dpi)				After P3 challenge (25 to 33 dpi/1 to 9 dpc)			
	P3 (n = 6)	P100C4 (*n* = 6)	P120 (*n* = 6)	Mock (*n* = 6)	P3 (*n* = 4)	P100C4 (*n* = 4)	P120 (*n* = 4)	Mock (*n* = 4)
PEDV diarrhea rate (%)	100	0	0	0	0	100	100	100
Mortality (%)	0	0	0	0	0	0	0	0
Duration of diarrhea (days, FC ≥ 2)	2.66 ± 1.37 ^a^	0.00 ± 0.00 ^b^	0.00 ± 0.00 ^b^	0.00 ± 0.00 ^b^	0.00 ± 0.00 ^c^	3.25 ± 0.50 ^b^	4.50 ± 0.58 ^a^	4.00 ± 0.82 ^a, b^
Cumulative FC scores	8.00 ± 1.79 ^a^	0.33 ± 0.52 ^b^	1.33 ± 2.00 ^b^	1.67 ± 0.98 ^b^	0.50 ± 1.00 ^c^	9.25 ± 0.50 ^b^	13.50 ± 1.73 ^a^	12.00 ± 0.82 ^a^
Highest viral shedding titer log_10_ N gene copies/ml)	11.63 ± 0.41	9.88 ± 2.71 ^*^	10.97 ± 1.61	NA	5.65 ± 1.59	11.75 ± 0.38	12.02 ± 0.64	12.28 ± 0.33
Duration of fecal PEDV RNA shedding (days)	12.83 ± 2.56 ^a^	2.00 ± 1.00 ^b^	1.17 ± 0.41 ^b^	NA	2.00 ± 1.92 ^c^	7.50 ± 1.00 ^b^	> 9 ^a^	> 9 ^a^
Weekly body weight gain (kg) ^**^	−0.3 ± 2.67 ^b^	1.83 ± 1.76 ^a^	2.28 ± 0.61 ^a^	1.90 ± 0.77 ^a^	2.79 ± 0.29 ^a^	3.98 ± 2.32 ^a^	3.55 ± 0.62 ^a^	− 0.45 ± 2.53 ^b^

^*^One pig in P100C4 group did not shed PEDV RNA in feces; ^**^ during the 1st week post-inoculation or challenge

^a, b, c^Different letters in each column denote differences among groups (*P* < 0.05)

dpi: days post-inoculation, dpc: days post-challenge, FC: fecal consistency (0, solid; 1, pasty; 2, semi-liquid; 3, liquid), N: nucleocapsid, NA: not available

No fecal PEDV RNA was detected in the pigs of the mock group (Fig. 1B). In the P3 group, all pigs shed medium-high PEDV RNA amounts (> 8 log_10_ copies/mL) in feces as early as 2 dpi. The mean titers peaked at 10.58 ± 0.99 log_10_ copies/mL at 3 dpi but dropped to a mean of 6.00 ± 2.94 at 4 dpi and 5.90 ± 2.69 log_10_ copies/mL at 5 dpi. Afterward, the mean titers peaked (10.81 ± 1.54 copies/mL) again at 6 dpi, decreased gradually and ceased by 19 dpi (11–19 dpi among pigs). Four of six (66.67%) pigs in the P100C4 group began shedding of fecal PEDV RNA at 5 dpi with mean titers of 10–12 log_10_ copies/ml, which were also the peak mean viral RNA titers of those pigs. However, the fecal viral RNA shedding was delayed in the fifth pig to 9 dpi and was not detected in the sixth pig. At 6, 9 and 11 dpi, individual pigs in the P100C4 group had low fecal PEDV RNA titers (4.8–7.5 log_10_ copies/mL) and stopped shedding by 13 dpi. All pigs in the P120 group shed fecal PEDV RNA with a mean titer peak (11.63 ± 0.41 log_10_ copies/mL) at 5 dpi and stopped shedding by 7 dpi.

### PEDV PC22A-P100C4 and -P120 elicited PEDV serum Ab responses in weaned pigs

**Fig. 2:**
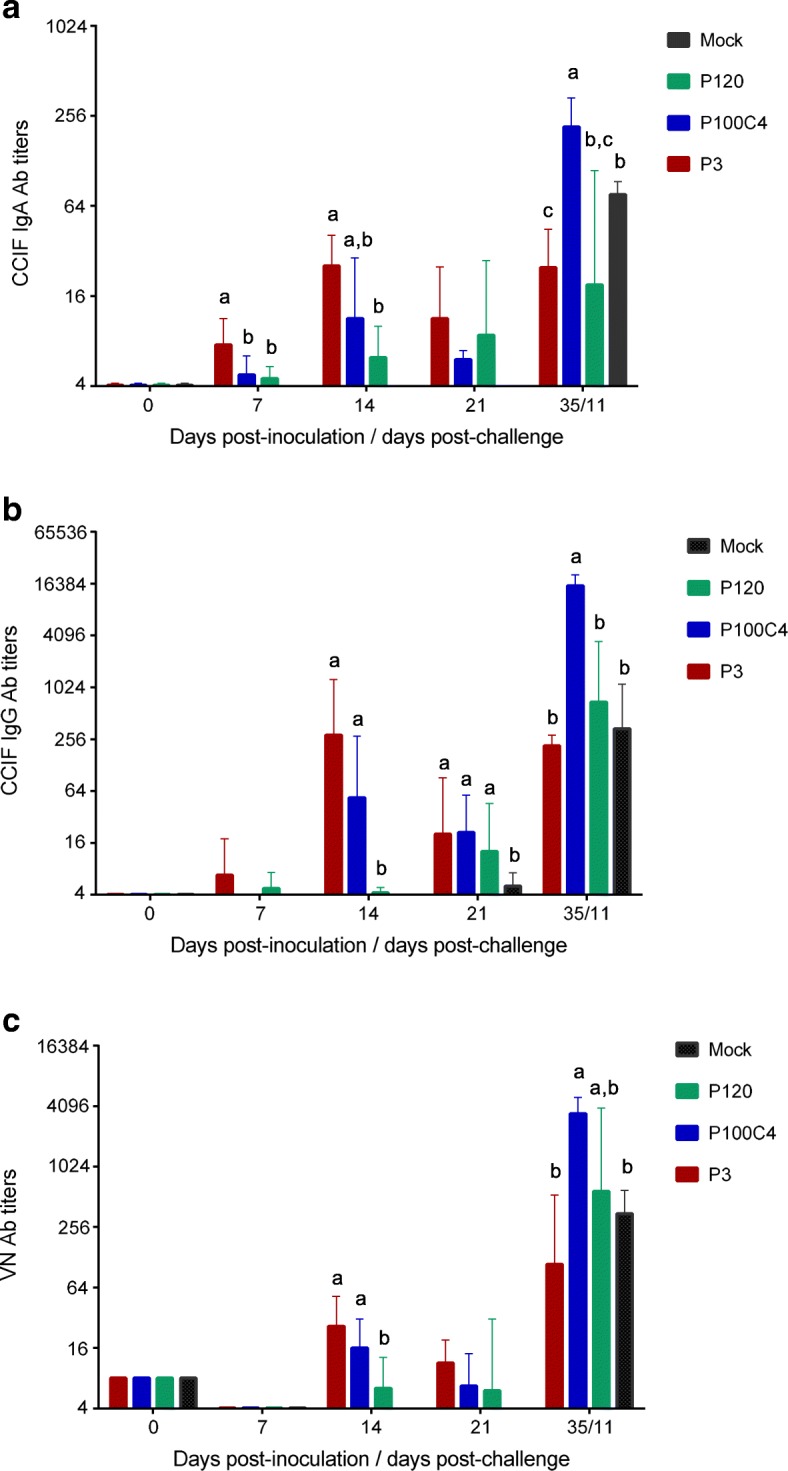
Serum IgA (**a**), IgG (**b**) and viral neutralizing (VN) (**c**) antibody titers to PEDV. Forty-day-old weaned pigs were inoculated with mock, P100C4, P120 or P3. Subsequently, all pigs were challenged with P3 at 24 days post-inoculation (dpi). Data are displayed as mean with standard deviation in each group. The numbers of animals in each treatment group were six at inoculation and four at the challenge. Different letters mean significant different levels among groups (P < 0.05)

The dynamics of serum PEDV-specific IgA and IgG Ab responses post inoculation among the P100C4, P120 and P3-inoculated pigs were similar, appeared in some pigs at 7 dpi, peaked at 14 dpi, and decreased at 21 dpi (Fig. 2). Serum IgG Ab titers were generally higher than IgA Ab titers at each time point. At 7 dpi, five and two of the six P3-inoculated pigs had detectable serum PEDV IgA and IgG Ab titers, respectively. All P3-inoculated pigs developed PEDV IgA and IgG Ab titers in serum by 14 dpi (Fig. 2A and B). Serum PEDV viral neutralization (VN) Abs were first detected at 14 dpi and the titers decreased at 21 dpi (Fig. 2C). Except for one pig that had no fecal viral RNA shedding and no PEDV Ab responses, the serum IgA, IgG and VN Ab titers in the P100C4 group were similar to those in the P3 group at 14 dpi. P120-inoculated pigs had no or very low titers of IgA, IgG or VN Ab after inoculation. No PEDV-specific Abs were detected in serum collected from pigs in the mock group. None of the pigs in this study had detectable serum PEDV-specific IgM Abs.

### PEDV PC22A P100C4- and P120-inoculation provided insufficient protection to pigs against virulent PC22A P3 challenge

After the challenge with P3, pigs in the P3 group had no clinical signs (Fig. 1A) and no or transient low to intermediate level of fecal PEDV RNA shedding (5.2 to 8.2 log_10_ copies/mL) (Fig. 1B). On the other hand, all pigs in mock, P120 and P100C4 groups had an acute onset of severe diarrhea (FC = 3) and high fecal PEDV RNA shedding titers. Cumulative FC scores from 1 to 9 dpc of P120 (13.50 ± 1.73) and mock (12.00 ± 0.82) groups were similar but significantly higher than that (9.25 ± 0.50) of the P100C4 group (*P* < 0.05) (Table 1). Fecal PEDV RNA shedding titers in mock, P120 and P100C4 groups peaked at a similar high level at 2 dpc/26 dpi. Subsequently, fecal PEDV RNA shedding titer in the P100C4 group decreased faster than those in the P120 and mock groups (Fig. 1B). The mean duration of fecal PEDV RNA shedding was 7.50 ± 1.00 days post challenge in the P100C4 group. However, pigs in the mock and P120 groups continuously shed PEDV RNA in feces until the end of this study. After the challenge, the pigs in the mock group had lost weight (Table 1), whereas the pigs in the P100C4 and P120 groups had similar body weight gain profiles to that in the P3 group.

### P100C4-inoculated pigs developed the highest secondary Ab responses among the three PEDV-inoculated groups after challenge with virulent P3 virus

The serum PEDV IgA, IgG and VN Ab titers of all groups of pigs increased after challenge (Fig. 2). The highest secondary Ab responses were observed in pigs of the P100C4 group whose serum PEDV Ab titers were significantly higher than those in the other three groups (*P* < 0.01) at 11 dpc/35 dpi (Fig. 2). On the other hand, the serum PEDV IgA and IgG Ab titers of pigs in the P3, P120, and mock groups were similar, except that pigs in the mock group had significantly higher serum PEDV IgA Ab titers than those in the P3 group (*P* < 0.05) (Fig. 2). Interestingly, the fold-increase of PEDV Ab in the P3 group (IgA: 2.83 ± 2.55, IgG: 24.76 ± 2.03, VN: 10.56 ± 4.14) were the same or lower than those in the mock group (IgA: 19.03 ± 1.22, IgG: 71.01 ± 4.44, VN: 86.82 ± 1.71), and the P120 group (IgA: 1.47 ± 1.65, IgG: 39.67 ± 3.36, VN: 152.22 ± 4.50), but consistently lower than those in the P100C4 (IgA: 38.05 ± 1.57, IgG: 407.31 ± 1.61, VN: 333.14 ± 1.35) group after challenge (Fig. 2).

**Fig. 3:**
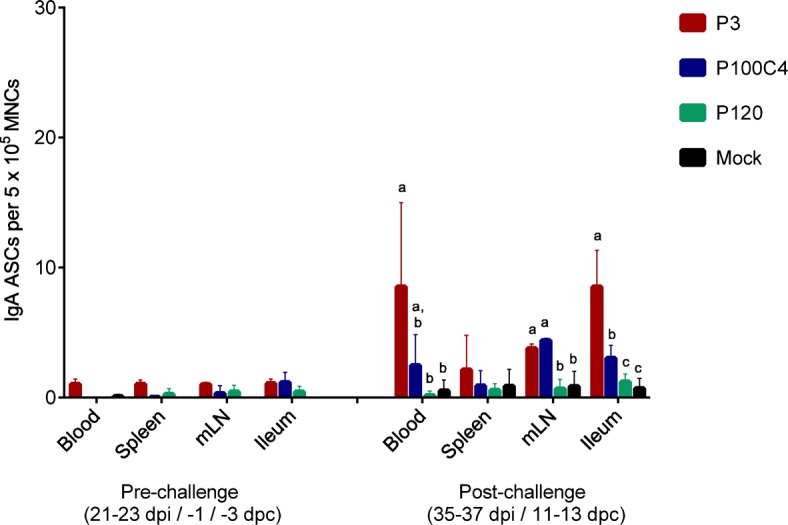
Numbers of IgA antibody-secreting cells pre- and post-challenge. The numbers of IgA antibody secreting cells (ASCs) per 5 × 10^5^ MNCs in blood, spleen, mesentery lymph nodes and ileum of mock-, P100C4-, P120- or P3-inoculated weaned pigs. Samples were collected at 21–23 days post-inoculation (dpi; before challenge; *n* = 2 per group) and at 35–37 dpi/11–13 days post-challenge (dpc; after challenge; n = 4 per group). Different letters indicate significantly different numbers of ASCs among the treatment groups (*p* < 0.05)

To investigate the intestinal Ab secretory cell responses, two and four pigs of each group were euthanized before challenge (at 21/23 dpi) and after challenge (at 11/13 dpc and 33/35 dpi), respectively. Before the challenge, PEDV-specific IgA Ab-secreting cells (ASCs) were detected in all pigs at low levels. At 11/13 dpc and 33/35 dpi, the P3 and P100C4 groups had a significantly higher number of IgA ASCs in the mesenteric lymph nodes (MLNs) and ileum (ranging from 2.4 to 8.5 per 5 × 10^5^ MSCs) than those in the P120 and mock groups (< 2 ASCs per 5 × 10^5^ MSCs) (*P* < 0.05) (Fig. 3). Raw data is provided in the Additional file 1.

## Discussion

A live attenuated PEDV vaccine should replicate in the gut, provide sufficient stimulation to elicit protective mucosal immunity, but cause no or negligible clinical signs. However, these requirements were usually mutually exclusive when attenuated vaccines were developed by using traditional cell culture methods alone. Previous studies reported that pigs orally or oronasally inoculated with attenuated PEDV [26], TEGV [27, 28] or rotavirus [29] strains developed lower protective immunity than pigs infected with the corresponding virulent virus. In TGEV naive sows, only oral inoculation with virulent TGEV or with multi doses of attenuated TGEV vaccines induced passive protection in the nursing pigs [10, 11]. In this study, pigs inoculated with virulent PEDV PC22A P3 developed complete protective immunity against subsequent challenge with a high dose of the homologous virus. Although one pig in the P120 group had diarrhea (FC = 2) at 1–2 dpi when its fecal PEDV RNA shedding was extremely low (4.9 log_10_ copies/mL at 1 dpi) or undetectable (at 2 dpi), diarrhea probably cannot be attributable to P120 infection. So, neither the attenuated PC22A-P100C4 nor the P120 caused disease in 40-day-old weaned pigs, fulfilling the safety requirement for a vaccine. On the other hand, one dose of P100C4 or P120 did not induce sufficient immunity to protect the pigs from diarrhea post challenge. The optimal stimulation of mucosal immune responses requires local virus replication and adequate amounts of viral antigens present for a sufficient period of time. The ability of the virus to replicate in intestinal epithelial cells and the dose of virus inoculum are two critical factors [17, 23]. The short duration of fecal PEDV RNA shedding indicates that P100C4 and P120 replicated to a more limited extent in weaned pigs than P3. The inoculum dose [6 log_10_ plaque formation unit (PFU) per pig] used for P100C4 and P120 viruses was similar to that (12 log_10_ copies/mL, corresponding to 6 log_10_ PFU) for the virulent PEDV PC21A strain, which was isolated from the same PEDV outbreak as the PC22A strain [30]. Whether a higher dose than the current 6 log_10_ PFU per pig and/or multiple doses of P100C4 and P120 would enhance the protection efficiency should be examined in the future.

In our previous study, we confirmed that P100C4 and P120 were partially attenuated in neonatal pigs [20]. P100C4- and P120-inoculated piglets had milder villous atrophy, delayed onset and shortened duration of fecal PEDV RNA shedding compared with P3-inoculated piglets. Furthermore, P120 was more attenuated than P100C4 based on longer incubation time, lower fecal virus shedding titers and more limited viral antigen distribution in the intestines. In this study, P3 was still virulent, and P100C4 and P120 were completely attenuated in weaned pigs, probably due to all age-dependent resistance to disease in weaned versus neonatal piglets [21, 22]. P100C4 and P120 did not show substantial differences in pathogenicity as neither P100C4 nor P120 caused clinical signs in weaned pigs. Besides, in one of the six pigs in P100C4 group productive infection was not established, suggesting that host-related variability in susceptibility to PEDV probably contributed to this result. However, these three viruses differ significantly in immunogenicity. In the P100C4 group, but not the P120 group, titers of serum PEDV IgA, IgG and VN Ab and the numbers of PEDV IgA ASCs were boosted significantly after the P3 challenge. In addition, fecal PEDV RNA shedding titers in the P100C4 group decreased earlier than those in the mock and P120 groups post challenge. These results suggest that P100C4 infection induced stronger primary and anamnestic immune responses. However, the P120-inoculated pigs had lower serum PEDV Ab titers than those inoculated with P3 or PC100C4 post inoculation. After the challenge with P3, anamnestic immune responses in the P120 group were marginal. Interestingly, serum secondary Ab responses in the P3 group were lower than those in the P100C4 group, although the number of ileum IgA ASCs was significantly higher in the P3 group than the P100C4 group. Because intestinal IgA Ab is generally considered as the most relevant to protection against PEDV infection [11, 17]. It is likely that pigs inoculated with virulent P3 developed sufficient intestinal mucosal immune responses to efficiently neutralize the challenge virus and prevent virus replication, corresponding with low fecal PEDV RNA shedding and no disease. On the other hand, even attenuated P100C4 elicited some IgA and IgG Ab response at 14 and 21 dpi, it did not provide weaned pigs with sufficient mucosal protection against virulent P3 challenge. So the replication of the challenge virus in pigs of the P100C4 group induced higher serum secondary immune responses than in the P3 group.

Two previous studies compared the immune responses induced by virulent and attenuated PEDV in 11-day-old and 5-week-old conventional pigs, respectively [23, 24]. In general, the virulent PEDV provided higher levels of protective immunity than the attenuated strains. None of the virulent PEDV-inoculated pigs developed clinical signs after challenge with the homologous virus. However, different attenuated viruses induced various protection rates. In the study of attenuated PEDV CV777 strain [23], 0, 12.5 and 46% of pigs in high dose group [5.4 × 10^6^ fluorescent focus formation unit (FFU)/pig], low dose group (2.55 × 10^5^ FFU/pig), and mock group, respectively, developed diarrhea after virulent CV777 challenge (unknown dose) at 21 dpi. In the study of attenuated PEDV PT strain [24], 100% (3/3) of mock-inoculated pigs developed watery diarrhea after challenge with 5.0 × 10^5^ TCID_50_/pig of virulent PEDV PT-P5 at 4 wks post inoculation, while none (0/6) of attenuated PEDV PT-P96-inoculated pigs developed diarrhea after challenge. In this study, all the pigs in the P100C4, P120, and mock groups showed diarrhea after challenge with the P3 virus (5.6 log_10_ PFU/pig) at 24 dpi. Overall, different virus strains and pig conditions (ages, sources of pigs, breed etc.) used by different laboratories may contribute to the variable protection rates. In addition, we did not investigate PEDV-specific cellular immune responses that may play an important role in protection against PEDV challenge. More studies on T cell immune responses to PEDV infection in the future will provide a more complete picture of protective immunity and better guidance for PEDV vaccine development.

The long-term goal of our study is to develop safe and efficacious live attenuated vaccines targeting sows to generate lactogenic immunity to protect neonatal piglets from PED. In this study, conventional weaned pigs were used to evaluate the safety and efficiency of the vaccine candidates because of three reasons: 1) previous studies suggest that young pigs can be used as a surrogate model to evaluate potential sow vaccines, 2) it is more economical compared with using sows to evaluate safety and immunogenicity of a vaccine candidate, and 3) weaned pigs are potential targets for vaccination for active immunity agaist PED [9]. Two partially attenuated PEDV vaccine candidates, PC22A-P100C4 and -P120, replicated to a lower extent than the P3 virus and caused no disease in weaned pigs. However, concerns remain because the attenuated PEDV strains may revert to virulence via mutation in the natural hosts or generate new virulent strains by exchanging their genes with field strains via recombination. With the development of reverse genetics technology [31, 32], virus with limited replication competency and defective in recombination can be engineered to alleviate this concern in the future. On the other hand, both PC22A-P100C4 and -P120 induced insufficient immune responses to protect pigs from the challenge with a high dose of virulent virus. After the challenge, only P100C4-, but not P120-, inoculated pigs developed strong anamnestic Ab responses. Considering the fact that feedback method using highly virulent PEDV viruses may facilitate the spread of other infectious agents in the herds and PEDV persistence on farms, and inactivated/subunit PEDV vaccine is mainly effective in previously PEDV-exposed but not PEDV-naive sows, a safe and efficacious live attenuated PEDV vaccine is still urgently needed. However, our results demonstrate the difficulties and challenges in the development of live attenuated vaccines using cell culture adaptation method alone. With the availability of an infectious clone system for PEDV [31], the P100C4 can be further modified to enhance its immunogenicity and maintain its attenuation. The combination of attenuated (such as P100C4) and inactivated vaccines, and/or the inclusion of mucosal adjuvants may be a safe and effective PEDV vaccination regimen as confirmed for TGEV sow vaccines [28]. All of these hypotheses can be investigated in the future.

## Conclusions

The Vero cell adapted PEDV PC22A P100C4 and P120 were fully attenuated in weaned pigs but failed to provide protection against diarrhea caused by the challenge with highly virulent PC22A P3. Our results demonstrated the difficulty in obtaining fully attenuated PEDV that retains its immunogenicity using traditional cell culture adaptation approaches. Therefore, understanding the molecular mechanisms of PEDV attenuation and rational design of vaccines using reverse genetics technology is the direction for the development of safe and efficacious attenuated vaccines for PEDV.

## Methods

### Viruses

The isolation and continuous passaging of PEDV PC22A strain in Vero cells were reported previously [20, 33]. The third passage (P3) of PC22A was used as the virulent inoculum (positive control) and the homologous challenge pool. The pathogenicity of P3 was confirmed as highly virulent: three PFU of P3 caused severe diarrhea and mortality in 100% (8/8) of 4-day-old Cesarean-derived, colostrum-deprived (CDCD) piglets [34]. Two high-level passages (P100 and P120) of PC22A were selected as attenuated vaccine candidates. P100 was plaque-purified and its clone no. 4 (P100C4) was used in this study. Both P100C4 and P120 caused moderate diarrhea in 75% (3/4) of CDCD piglets without mortality. Detailed information regarding the virus growth kinetics in cell culture, virulence in neonatal pigs, and the whole genomic sequence analysis was reported previously [20].

### Animals

Twenty-four conventional weaned pigs at 28 days of age were purchased from The Ohio State University (OSU) specific pathogen-free (SPF) herd that has no history of PEDV, TGEV, or porcine reproductive and respiratory syndrome virus (PRRSV) outbreaks. Nasal and rectal swabs were collected from all pigs and confirmed as negative for PEDV, TGEV/porcine respiratory coronavirus (PRCV), porcine delta coronavirus, and porcine group A and group C rotaviruses as described previously [30]. Conventional RT-PCR [35] revealed a weak positive for group A porcine rotavirus (data not shown). After 12 days of acclimation in our biosafety level 2 (BSL2) facilities, at 40 days of age, no pig had diarrhea and their rectal swab samples were negative for rotavirus RNA by RT-PCR.

### Experimental design of weaned pig studies

Twenty-four 40-day-old weaned pigs were randomly divided into four groups, with six pigs/group. The initial mean body weight of pigs in each group was similar. Each group of pigs was housed in a separate room and orally inoculated with 5 log_10_ PFU of P3, 6 log_10_ PFU of P100C4, 6 log_10_ PFU of P120, or virus-free medium (mock infection), respectively (Table 1). The oral inoculum doses were based on our previous data that 11–12 log_10_ nucleocapsid (N) gene copies) of viral RNA, corresponds to approximately 5–6 log_10_ PFU of highly virulent US PEDV PC21A strain was required to infect 32-day-old conventional pigs [30]. After inoculation, piglets were observed twice daily for clinical signs, including vomiting, diarrhea, and anorexia. Rectal swabs were collected daily for the first 7 dpi and for every other day thereafter. Fecal consistency (FC) was scored as follows: 0, solid; 1, pasty; 2, semi-liquid; 3, liquid, respectively. A fecal consistency score of ≥2 was considered as diarrhea. Fecal PEDV RNA shedding was detected by TaqMan real-time reverse transcription-PCR (RT-qPCR) assay [33]. Body weights were measured and blood samples were obtained before inoculation and weekly post-inoculation. At 21–23 dpi, 2 pigs per group were randomly selected and euthanized to examine systemic and intestinal IgA ASCs. At 24 dpi all pigs were orally challenged with a virulent P3 virus. Considering the clinical signs of P3-inoculated pigs and the fact that challenge was done in older pigs at 24 dpi, the dose used for challenge was increased to 5.6 log_10_ PFU/pig, which was higher than that (5.0 log_10_ PFU/pig) used for the initial inoculation of pigs in the P3 group. FC score was recorded until 3 days after no pig had diarrhea. The study was terminated at 35–37 dpi/11–13 days post-challenge (dpc) when all pigs were euthanized for blood and tissue collection. The pigs were euthanized with TKX combo [2.5 mL xylazine (100 mg/mL) and 2.5 mL Ketamine (100 mg/mL) in Telazol (tiletamine 10 mg/mL and zolazemine 10 mg/mL)] at a dose of 0.05 mL/kg weight (intramuscular injection) followed by electrocution and exsanguination.

### PEDV RT-qPCR for the detection of viral RNA in feces

RS samples were suspended in Minimum Essential Media (Invitrogen, Carlsbad, CA, USA) as a 10% fecal suspension. The RNA was extracted using the MagMax™-96 Viral Isolation kit (Ambion, Austin, TX, USA) then subjected to RT-qPCR with the primers and probe targeting the PEDV N gene [33]. The detection limit was 10 N gene copies per 20 μL of reaction, corresponding to 4.8 log_10_ copies per mL of original fecal sample.

### Cell culture immunofluorescence assay for determination of the serum PEDV IgA and IgG Ab titers

Monolayers of Vero cells in 96 well-plates were inoculated with PC22A-P10 overnight and fixed with acetone-methanol (20:80) at − 20 °C for 10 min. Serial 4-fold dilutions of pig serum samples in PBS were prepared. Fifty-microliters of the sample dilutions were added to each well. The plates were incubated at room temperature for 1 h and then rinsed twice with PBS for 10 min. Fluorescein isothiocyanate (FITC)-conjugated goat anti-swine IgG (KPL, West Chester, PA) or goat anti-swine IgA (BioRad, Hercules, CA) at a 1:200 or 1:500 dilution was then added. After 1 h of incubation in the dark, plates were rinsed twice with PBS for 5 min each time and examined using a fluorescence microscope (Olympus, Center Valley, PA). For each test, one positive antiserum with known PEDV Ab titer and one negative pig serum were included as positive and negative controls, respectively. All samples were tested at least in triplicate. The Ab titer was expressed as the mean of repeats and calculated based on the log_2_ transformation.

### Fluorescent focus reduction virus neutralization (FFRVN) assay

Detailed procedures for the FFRVN were described previously [36]. Briefly, pig serum samples were inactivated at 56 °C followed by serial 4-fold dilutions in PBS. 50 μl of each dilution was then mixed with an equal volume of PEDV PC22A-P10 stocks containing 100 fluorescent focus units (FFU). After 1 h incubation at 37 °C, the serum-virus mixtures were inoculated onto confluent Vero cell monolayers. To enhance virus absorption, a brief centrifugation (1000×g for 10 min) was conducted. Subsequently, the inoculum was removed and replaced by 100 μl of DMEM supplemented with 5 μg/ml trypsin. After 4 h incubation, 100 μl of DMEM supplemented with 5% FBS was added to block the action of trypsin and the plates were incubated overnight. The cell culture immunofluorescence assay (CCIF) procedure was then conducted using PEDV N monoclonal Ab (SD6–29) and FITC-conjugated goat anti-mouse IgG (Serotec) as primary and secondary antibodies, respectively. The numbers of FFUs in the wells were counted and the VN Ab titer of each serum sample was expressed as the reciprocal of the dilution giving an 80% reduction in the number of FFU. All sera were tested in triplicate, and the final Ab titer was expressed as the mean of repeats.

### Mononuclear cell (MNC) isolation

The MNCs from the spleen, MLN, ileum, and blood were isolated by following routine procedures in our laboratory [37]. Fragments of ileum were washed twice, first with washing solution (RPMI 1640 with 10 mM HEPES [N-2-hydroxyethylpiperazine-N9–2-ethanesulfonic acid], 200 mg of gentamicin per mL, and 20 mg of ampicillin per mL) followed by Hanks’ balanced salt solution. The tissues were cut into small pieces, placed in HBSS containing 1 mM dithiothreitol and 5 mM EDTA, and vigorously shaken for 30 min to dislodge the epithelial cells. Subsequently, the segments were minced, suspended in RPMI 1640 containing 10% fetal bovine serum and 300 U of type II collagenase (Sigma Chemical Co., St. Louis, Mo.) per mL for digestion at 37 °C for 30 min with gentle shaking. Afterward, the supernatants were collected, and the remaining tissues were pressed through stainless steel Collectors fitted with an 80 μm mesh screen (Collector, FL, USA). Similarly, spleen and MLN were also minced and pressed through stainless steel 80-mesh screens. Single cellular suspensions were then collected and subjected to gradient centrifugation in Percoll (Sigma). Blood was collected aseptically in 25% (*v*/v) acid citrate glucose. The peripheral blood lymphocytes were isolated by Ficoll-Paque (Ficoll-Paque Research Grade, Pharmacia Biotech., Uppsala, Sweden) density gradient centrifugation. Collected MNCs were subjected for frozen in liquid nitrogen as described [38].

### ELISpot assay for the detection of PEDV-specific IgA ASCs

Confluent Vero cell monolayers in 96-well plates were inoculated with PEDV PC22A P10 at MOI = 0.1. At 18 hpi, over 90% of cells were infected with PEDV, tested by CCIF, and the cell monolayer was still intact. Plates were fixed with 80% acetone for 20 min, air-dried and stored at − 20 °C until use.

On the day of assay, MNCs were quickly thawed from liquid nitrogen by first resuspending in cold E-PMI followed by centrifugation and removal of the DMSO in the freezing medium. Then the cells were resuspended in E-PMI and incubated at 37 °C with 5% CO_2_ for 4 h. The plates were thawed and rehydrated by incubation with PBS at room temperature. Different amounts of live MNCs (5 × 10^5^, 5 × 10^4^, 5 × 10^3^ and 5 × 10^2^) from each tissue were added to duplicate wells. Plates were centrifuged at 120 x g for 5 min and incubated for 16 h at 37 °C with 5% CO_2_. Subsequently, the plates were washed with PBS containing 0.05% Tween 20 (PBST). The Ab production was detected using horseradish peroxidase-labeled affinity-purified goat anti-pig IgA antibodies (KPL, Maryland) diluted 1:4000 in PBST and incubated for 2 h at RT. Finally, the spots were developed by tetramethylbenzidine (TMB) with H_2_O_2_ membrane peroxidase substrate system (KPL). Counts were averaged from duplicate wells. Data were expressed as the mean number of ASCs per 5 × 10^5^ MNCs [37].

### Statistical analysis

Analysis of variance (ANOVA) followed by Duncan’s multiple-range test was used to demonstrate statistical differences in the virus shedding/Ab titers and number of ASCs among the four pig groups. The FC scores were compared using Kruskal-Wallis non-parametric analysis. To compare the body weight gain among groups, analysis of covariance (ANCOVA) was applied to adjust for initial body weights. Comparison of piglets’ serum Ab titers at each time point was conducted using a paired t-test. Statistical analyses were done using SAS (Statistical Analysis System; SAS for windows 9.12; SAS Institute Inc., Cary, NC, USA). A *p* value of < 0.05 was considered significant.

## Additional file

**Additional file 1:** Raw data of fecal consistency, fecal PEDV RNA shedding titers, serum antibody titers, and ELISpot of the four groups of pigs. (XLSX 21 kb)

## Abbreviations

Ab: antibody
ANCOVA: analysis of covariance
ANOVA: analysis of variance
ASCs: antibody secreting cells
CCIF: Cell culture immunofluorescence assay
CDCD: Cesarean-derived, colostrum-deprived
DPC: day(s) post-challenge
DPI: day(s) post-inoculation
ELISpot: enzyme-linked immunospot
FC: Fecal consistency
FFRVN: Fluorescent focus reduction virus neutralization
IFA: immunofluorescence assay
MLNs: mesenteric lymph nodes
N: nucleocapsid
PBS: phosphate buffered saline
PED: porcine epidemic diarrhea
PEDV: porcine epidemic diarrhea virus
PFU: plaque formation unit
RT-qPCR: TaqMan real-time reverse transcription-PCR
SPF: specific pathogen-free
TCID_50_: 50% tissue culture Infectious dose
TGE: transmissible gastroenteritis
TGEV: transmissible gastroenteritis virus
VN: viral neutralization/neutralizing
